# Achieving low-density lipoprotein cholesterol targets as assessed by different methods in patients with familial hypercholesterolemia: an analysis from the HELLAS-FH registry

**DOI:** 10.1186/s12944-020-01289-5

**Published:** 2020-05-28

**Authors:** Christos V. Rizos, Matilda Florentin, Ioannis Skoumas, Konstantinos Tziomalos, Loukianos Rallidis, Vasileios Kotsis, Vasileios Athyros, Emmanouil Skalidis, Genovefa Kolovou, Anastasia Garoufi, Eleni Bilianou, Iosif Koutagiar, Dimitrios Agapakis, Estela Kiouri, Christina Antza, Niki Katsiki, Evangelos Zacharis, Achilleas Attilakos, George Sfikas, Panagiotis Anagnostis, Demosthenes B. Panagiotakos, Evangelos N. Liberopoulos

**Affiliations:** 1 Department of Internal Medicine, Faculty of Medicine, University of Ioannina, Ioannina, Greece; 2 1st Department of Cardiology, Medical School, National and Kapodistrian University of Athens, Hippokration Hospital, Athens, Greece; 3 1st Propedeutic Department of Internal Medicine, Medical School, Aristotle University of Thessaloniki, AHEPA Hospital, Thessaloniki, Greece; 4 Department of Cardiology, Medical School, National and Kapodistrian University of Athens, Attikon University General Hospital, Athens, Greece; 5 Department of Internal Medicine, Medical School, Aristotle University of Thessaloniki, Papageorgiou General Hospital Thessaloniki, Thessaloniki, Greece; 6 Department of Internal Medicine, Medical School, Aristotle University of Thessaloniki, Hippokration General Hospital, Thessaloniki, Greece; 7 Cardiology Clinic, University General Hospital of Heraklion, Heraklion, Greece; 8 Cardiology Clinic, Onassis Cardiac Surgery Center, Athens, Greece; 9 Department of Pediatrics, Medical School, National and Kapodistrian University of Athens, 2nd Pediatrics Clinic, General Children’s Hospital “Pan. & Aglaia Kyriakou”, Athens, Greece; 10 Cardiology Clinic, “Tzaneio” General Hospital, Piraeus, Greece; 11 Department of Pediatrics, Medical School, National and Kapodistrian University of Athens, C’ Pediatrics Clinic, Attikon University General Hospital, Athens, Greece; 12 Department of Internal Medicine, 424 Military Hospital, Thessaloniki, Greece; 13 Department of Endocrinology, Police Medical Center of Thessaloniki, Thessaloniki, Greece; 14 Department Of Nutrition & Dietetics, School of Health Science & Education, Harokopio University, 70 Eleftheriou Venizelou (Thiseos) Ave, Kallithea, 176 71 Athens, Greece

**Keywords:** Familial hypercholesterolemia, Greece, Hellenic familial hypercholesterolemia registry, Cardiovascular disease, Hypolipidemic treatment, Target achievement, Friedewald, Martin/Hopkins, Lipoprotein (a)

## Abstract

**Background:**

Familial hypercholesterolemia (FH) is characterized by elevated low-density lipoprotein cholesterol (LDL-C) levels and increased cardiovascular disease (CVD) risk. FH patients often have increased lipoprotein(a) [Lp(a)] levels, which further increase CVD risk. Novel methods for accurately calculating LDL-C have been proposed.

**Methods:**

Patients with FH were recruited by a network of Greek sites participating in the HELLAS-FH registry. LDL-C levels were calculated using the Friedewald (LDL-C_F_) and the Martin/Hopkins (LDL-C_M/H_) equations as well as after correcting LDL-C_M/H_ for Lp(a) levels [LDL-C_Lp(a)corM/H_]. The objective was to compare LDL-C levels and target achievement as estimated by different methods in FH patients.

**Results:**

This analysis included 1620 patients (1423 adults and 197 children). In adults at diagnosis, LDL-C_F_ and LDL-C_M/H_ levels were similar [235 ± 70 mg/dL (6.1 ± 1.8 mmol/L) vs 235 ± 69 mg/dL (6.1 ± 1.8 mmol/L), respectively; *P* = NS], while LDL-C_Lp(a)corM/H_ levels were non-significantly lower than LDL-C_F_ [211 ± 61 mg/dL (5.5 ± 1.6 mmol/L); *P* = 0.432]. In treated adults (*n* = 966) both LDL-C_F_ [150 ± 71 mg/dL (3.9 ± 1.8 mmol/L)] and LDL-C_M/H_ levels [151 ± 70 mg/dL (6.1 ± 1.8 mmol/L); *P* = 0.746] were similar, whereas LDL-C_Lp(a)corM/H_ levels were significantly lower than LDL-C_F_ [121 ± 62 mg/dL (3.1 ± 1.6 mmol/L); *P* < 0.001]. Target achievement as per latest guidelines in treated patients using the LDL-C_M/H_ (2.5%) and especially LDL-C_Lp(a)corM/H_ methods (10.7%) were significantly different than LDL-C_F_ (2.9%; *P* < 0.001).

In children, all 3 formulas resulted in similar LDL-C levels, both at diagnosis and in treated patients. However, target achievement by LDL-C_F_ was lower compared with LDL-C_M/H_ and LDL-C_Lp(a)corM/H_ methods (22.1 vs 24.8 vs 33.3%; *P* < 0.001 for both comparisons).

**Conclusion:**

LDL-C_Lp(a)corM/H_ results in significantly lower values and higher target achievement rate in both treated adults and children. If validated in clinical trials, LDL-C_Lp(a)corM/H_ may become the method of choice to more accurately estimate ‘true’ LDL-C levels in FH patients.

## Background

Familial hypercholesterolemia (FH) is the most common inherited metabolic disease, with around 13 million people worldwide being affected [1]. FH is characterized by markedly elevated low-density lipoprotein cholesterol (LDL-C) levels. This results in increased atherosclerotic burden in FH patients. Indeed, FH patients have been shown to have increased intima-media thickness and increased coronary artery disease prevalence [2, 3]. Moreover, FH patients with acute coronary syndrome have significantly higher all-cause mortality at 36- and 60-month follow-up in comparison to non-FH subjects [4]. Therefore, FH patients should be aggressively managed, and their co-morbidities comprehensively addressed combining hypolipidemic medications and dietary intervention [5–7]. Evidence suggests that < 5% of FH patients are diagnosed, with higher detection rates in countries with formal screening programs; among those diagnosed, only 10–25% are appropriately treated [1]. Even with optimal available therapies, about 80% of patients do not reach guideline-recommended LDL-C goals [1, 8].

The most widely used equation for LDL-C estimation is the Friedewald formula, which obviates the need for ultracentrifugation [9]. This equation utilizes the fixed value of 5 for the ratio of triglycerides (TG) to very low-density lipoprotein cholesterol (VLDL-C); however, there is great variance in this ratio across the range of TG and non-high density lipoprotein cholesterol (non-HDL-C) levels [10]. Martin et al. developed and validated a different method for estimating LDL-C, using an adjustable factor for the TG:VLDL-C ratio based on TG and non-HDL-C concentrations [11]. Nevertheless, calculated LDL-C includes the cholesterol of lipoprotein(a) [Lp(a)-C] [12], irrespectively of the method used, as none of them can separate ‘true’ LDL-C from Lp(a)-C [13, 14]. Of note, this is also the case for direct LDL-C assays and ultracentrifugation [15]. The main reason stems from the fact that LDL and Lp(a) significantly overlap in densities, with the presence of LDL in density ranging from 1.019 to 1.063 g/mL and Lp(a) from 1.040 to 1.21 g/mL, respectively [16, 17]. In this context, Dahlen modified the Friedewald equation in order to account for Lp(a) [i.e. calculating LDL-C corrected for Lp(a)] [18].

In the present study, we compared LDL-C levels as assessed with the Friedewald (LDL-C_F_) and Martin/Hopkins (LDL-C_M/H_) equations, as well as after correcting LDL-C_M/H_ for Lp(a) concentrations [LDL-C_Lp(a)corM/H_] in patients participating in the Hellenic FH (HELLAS-FH) Registry. We also compared the percentage rate of LDL-C treatment targets, as proposed by the 2019 European Society of Cardiology/European Atherosclerosis Society (ESC/EAS) guidelines, with each equation [19]. A separate analysis was performed for children.

## Methods

### Study design

The design and rationale of the HELLAS-FH registry have been previously described [20, 21]. In brief, HELLAS-FH registry is based on a network of sites that are distributed throughout Greece. Patients with FH are enrolled in an electronic database after signing an informed consent form. For the diagnosis of FH in adults the Dutch Lipid Clinic Network (DLCN) criteria are used, which have been shown to have an 85% agreement rate with the genetic diagnosis [22, 23]. Patients with at least a possible diagnosis of FH (DLCN score ≥ 3) are enrolled in the registry. Regarding the diagnosis of children with FH, the current EAS consensus statement is used [24]. LDL-C levels were calculated by the Friedewald formula: LDL-C_F_ = Total Cholesterol (TCHOL) - TG/5 - HDL-C. Martin et al. developed and validated a novel method for estimating LDL-C by using data from 1,310,440 patients [11]. Cholesterol concentrations including LDL-C, VLDL-C and HDL-C were directly measured by ultracentrifugation. Triglycerides were directly measured using the ARCHITECT C-8000 system (Abbott). This allowed direct comparison of TG levels to VLDL-C levels in every individual in the dataset. For stratification, they used TG and non-HDL-C because of their performance in explaining variance in the TG:VLDL-C ratio compared with other combinations of parameters and because they capture information on the 3 core elements from the standard lipid profile. Varying the number of TG and non-HDL-C strata based on quantiles or accepted cut points, they generated a 2-dimensional table of median TG:VLDL-C ratios using 180 cells. As a result an adjustable factor for the TG:VLDL-C ratio based on TG and non-HDL-C concentrations was created [11]. The new algorithm to estimate LDL-C was: LDL-C_M/H_ = TCHOL - TG/(adjustable factor) - HDL-C, where the adjustable factor stood for the strata-specific median TG:VLDL-C ratio. Moreover, the LDL-C corrected for Lp(a) levels was calculated by applying the Dahlen correction on the LDL-C_Μ/Η_ [LDL-C_Lp(a)corM/H_ = LDL-C_M/H_ - 0.3*Lp(a)].

Biochemical parameters were measured at the local laboratory of each site after an overnight fast by standard methods across different laboratories. Serum concentrations of TCHOL and TGs were determined enzymatically, HDL-C was determined by a direct assay and Lp(a) was measured by immunonephelometry.

### Statistical analysis

Continuous variables were tested for lack of normality by the Kolmogorov-Smirnov test. Values are expressed as mean ± standard deviation (SD) and median [interquartile range (IQR)] for variables with and without normal distribution, respectively. Characteristics of the study population are presented as frequencies and percentages for categorical variables. The Student’s paired t-test was used to compare different results between the different LDL-C calculating formulas. Pearson’s correlation was carried out between LDL-C_F_ and LDL-C_M/H_ as well as LDL-C_Lp(a)corM/H_. The differences between estimated LDL-C levels, as assessed by the 3 methods, were tested using the Wilcoxon matched-pairs signed rank test in each of the quartiles of LDL-C_F_. A probability value of *P* < 0.05 was considered statistically significant, and all probability values were 2-sided. Analyses were performed using the Statistical Package for the Social Sciences (SPSS) 21.0 (SPSS Inc., Chicago, IL).

## Results

### Adult subgroup

A total of 1423 adult patients (733 males) were included in the analysis. The mean age of the population at the time of enrolment was 51.3 ± 14.3 years and at FH diagnosis 44.3 ± 15.8 years. The median DLCN score was 5 (4–8). Baseline demographic characteristics are presented in Table 1 and lipid profiles in Table 2.

**Table 1:** Baseline characteristics of familial hypercholesterolemia (FH) patients in the HELLAS-FH registry

		Adults	Children
**Number of patients**		1423	197
**Gender (male/female)**		733/690	100/97
**Age at registration (years)**		51.3 ± 14.3	11.0 ± 3.5
**Age at diagnosis (years)**		44.3 ± 15.8	7.2 ± 3.9
**DLCN score**		5 (4–8)	–
**Systolic blood pressure (mmHg)**		128 ± 14	111 ± 13
**Diastolic blood pressure (mmHg)**		77 ± 9	65 ± 9
**Heart rate (bpm)**		74 ± 10	79 ± 13
**Prevalence of distinctive clinical findings (%)**	***Corneal arcus below the age of 45 years***	8.3%	1.0%
	***Tendon xanthomas***	5.7%	2.6%
	***Xanthelasma***	5.9%	1.0%
**Body mass index (kg/m**^**2**^**)**		27.0 (24.2–29.7)	20.0 (17.4–22.4)
**Hypertension (%)**		28.1	0.0
**Type 2 diabetes (%)**		7.8	0.0
**Waist circumference**	***Male (cm)***	95 (88–103)	70 (61–80)
	***Female (cm)***	88 (80–98)	65 (57–80)
	***Men > 102 cm (%)***	26.6	–
	***Women > 88 cm (%)***	49.7	–
**Smokers (%)**	***Active***	24.7	–
	***Former***	9.7	–
	***Passive***	1.9	–
	***Never***	63.6	–

Values are expressed as number of patients, percentage of patients, mean ± standard deviation or median (interquartile range)

**Table 2:** Lipid profile of patients before initiation of any hypolipidemic treatment

Parameter	Adults		Children	
	At diagnosis (***n*** = 1423)	On treatment (***n*** = 966)	At diagnosis (***n*** = 197)	On treatment (***n*** = 117)
Total cholesterol, mg/dL (mmol/L)	318 ± 80 (8.2 ± 2.1)	228 ± 75^§^ (5.9 ± 1.9)	305 ± 64 (7.9 ± 1.7)	253 ± 98^§^ (6.5 ± 2.5)
Triglycerides, mg/dL (mmol/L)	130 (97–181) [1.5 (1.1–2.0)]	111 (80–157)^§^ [1.3 (0.9–1.8)]^§^	68 (59–89) [0.8 (0.7–1.0)]	111 (80–157) [1.3 (0.9–1.8)]
HDL-C, mg/dL (mmol/L)	51 ± 17 (1.3 ± 0.4)	51 ± 17 (1.3 ± 0.4)	57 ± 15 (1.5 ± 0.4)	51 ± 17 (1.3 ± 0.4)
non-HDL-C, mg/dL (mmol/L)	267 ± 81 (6.9 ± 2.1)	176 ± 75^§^ (4.6 ± 1.9)	248 ± 66 (6.4 ± 1.7)	176 ± 75^§^ (4.6 ± 1.9)^§^
LDL-C_F_, mg/dL (mmol/L)	235 ± 70 (6.1 ± 1.8)	150 ± 71^§^ (3.9 ± 1.8)	233 ± 65 (6.0 ± 1.7)	183 ± 97^§^ (4.7 ± 2.5)^§^
LDL-C_M/H_, mg/dL (mmol/L)	235 ± 69 (6.1 ± 1.8)	151 ± 70^§^ (3.9 ± 1.8)	229 ± 65 (5.9 ± 1.7)	180 ± 97^§^ (4.7 ± 2.5)^§^
^a^LDL-C_Lp(a)corM/H_, mg/dL (mmol/L)	211 ± 61 (5.5 ± 1.6)	121 ± 62^†,§^ (3.1 ± 1.6)	225 ± 70 (5.8 ± 1.8)	174 ± 94^†,§^ (4.5 ± 2.4)^§^
^a^Lp(a), mg/dL (nmol/L)	21 (9–56) [42.0 (15.8–118.3)]	20 (9–55) [42.0 (15.8–118.3)]	15 (8–50) [28.9 (13.6–105)]	20 (10–60) [39.8 (18.0–127.0)]
^a^Apolipoprotein AI, mg/dL (g/L)	143 ± 35 (1.4 ± 0.4)	144 ± 25 (1.4 ± 0.3)	140 ± 24 (1.4 ± 0.2)	144 ± 25 (1.4 ± 0.3)
^a^Apolipoprotein B, mg/dL (g/L)	156 ± 65 (1.6 ± 0.7)	110 ± 35^§^ (1.1 ± 0.4)	147 ± 36 (1.5 ± 0.4)	110 ± 35^§^ (1.1 ± 0.4)^§^

Data are presented as mean ± standard deviation or median (interquartile range) for parametric and non-parametric variables, respectively

*HDL-C* High-density lipoprotein cholesterol, *LDL-C*_*F*_ Low-density lipoprotein cholesterol as calculated by the Friedewald formula, *LDL-C*_*M/H*_ low-density lipoprotein cholesterol as calculated by the Marin/Hopkins formula, *LDL-C*_*Lp(a)corM/H*_ Corrected for Lp(a) levels low-density lipoprotein cholesterol as calculated by the Marin/Hopkins formula, Lp(a) was converted using the formula: Lp(a) nmol/L = 2.18 × Lp(a) mg/dL − 3.83

^†^: *P* < 0.001 vs LDL-C_F_, ^§^*P* < 0.001 vs diagnosis

^a^: Data available for 355 patients

**Fig. 1:**
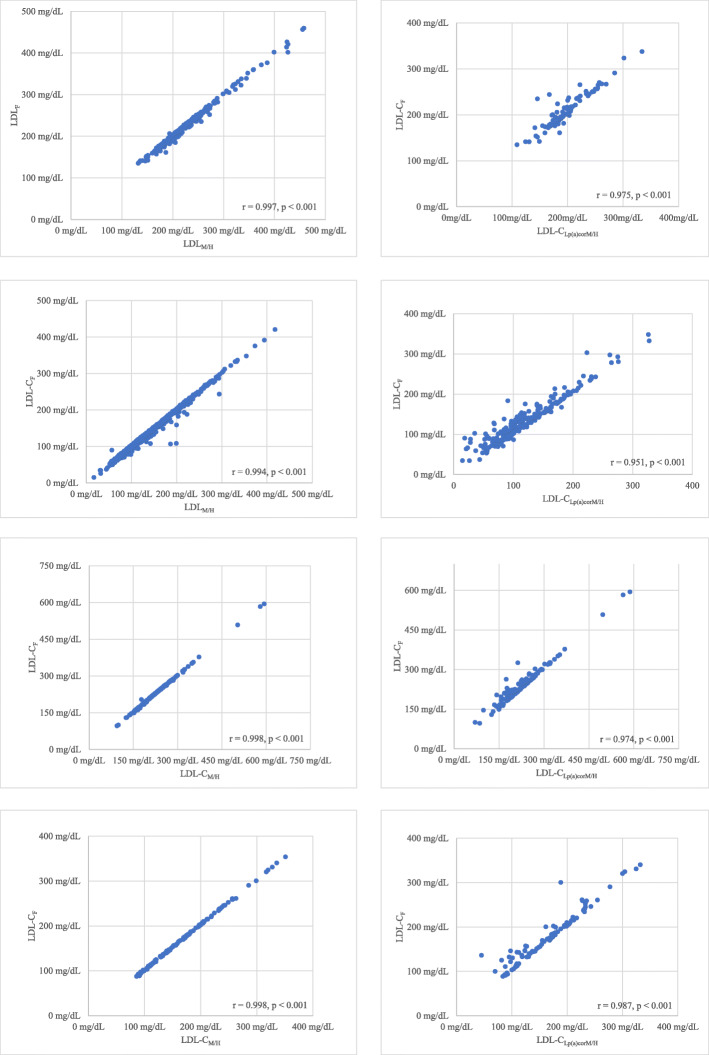
**a** Scatter correlation plot of pre-treatment LDL-C_F_ and LDL-C_M/H_ in adults. **b** Scatter correlation plot of pre-treatment LDL-C_F_ and LDL-C_Lp(a)corM/H_ in adults. **c** Scatter correlation plot of post-treatment LDL-C_F_ and LDL-C_M/H_ in adults. **d** Scatter correlation plot of post-treatment LDL-C_F_ and LDL-C_Lp(a)corM/H_ in adults. **e** Scatter correlation plot of pre-treatment LDL-C_F_ and LDL-C_M/H_ in children. **f** Scatter correlation plot of pre-treatment LDL-C_F_ and LDL-C_Lp(a)corM/H_ in children. **g** Scatter correlation plot of post-treatment LDL-C_F_ and LDL-C_M/H_ in children. **h** Scatter correlation plot of post-treatment LDL-C_F_ and LDL-C_Lp(a)corM/H_ in children

We first examined the lipid profile of adult FH patients at diagnosis. The LDL-C levels as calculated by the Friedewald [LDL-C_F_ = 235 ± 70 mg/dL (6.1 ± 1.8 mmol/L)] or the Martin/Hopkins formula [LDL-C_M/H_ = 235 ± 69 mg/dL (6.1 ± 1.8 mmol/L)] yielded similar results (*P* = 0.905) (Table 2). In patients with available Lp(a) concentrations (*n* = 355), LDL-C_Lp(a)corM/H_ was numerically lower [LDL-C_Lp(a)corM/H_ = 211 ± 61 mg/dL (5.5 ± 1.6 mmol/L)] than LDL-C_F_, but this difference did not reach significance (*P* = 0.432). Pearson’s correlation showed significant association between LDL-C_F_ and LDL-C_M/H_ (*r* = 0.997, *P* < 0.001, Fig. 1a) as well as between LDL-C_F_ and LDL-C_Lp(a)corM/H_ (*r* = 0.975, *P* < 0.001, Fig. 1b). Patients were split into quartiles according to pre-treatment LDL-C_F_ and the differences of LDL-C in each quartile were then compared across 3 methods (Table 3). Median LDL-C_M/H_ and LDL-C_Lp(a)corM/H_ were significantly lower compared with LDL-C_F_ in all but the first quartiles.

**Table 3:** Differences in LDL-C between the 3 methods based on pretreatment LDL-C_F_ quartiles

	LDL-C_F_ Quartile min-max	Pre-treatment (mg/dL)	
		dLDL_F-M/H_	dLDL_F-Lp(a)corM/H_
Adults	1st (132–196 mg/dL)	0.5 (−2.7–2.1)	2.2 (1.6–3.6)
	2nd (196–220 mg/dL)	1.6 (−0.7–2.5)^*^	6.5 (4.0–15.5)^*^
	3rd (220–258 mg/dL)	1.6 (−0.7–2.6)^*^	11.3 (5.9–19.2)^*^
	4th (258–680 mg/dL)	2.1 (0.0–3.0)^*^	9.9 (4.8–22.1)^*^
Children	1st (100–193 mg/dL)	2.6 (1.8–3.8)	3.8 (2.0–18.2)
	2nd (198–219 mg/dL)	3.9 (3.3–4.7)^*^	10.4 (5.8–24.6)^*^
	3rd (221–257 mg/dL)	3.8 (3.0–5.0)^*^	7.8 (5.5–9.7)^*^
	4th (258–594 mg/dL)	3.1 (2.6–3.8)^*^	8.3 (5.4–17.8)^*^

Values are presented as median (interquartile range)

*LDL-C*_*F*_ Low-density lipoprotein cholesterol as calculated by the Friedewald formula, *LDL-C*_*M/H*_ Low-density lipoprotein cholesterol as calculated by the Marin/Hopkins formula, *LDL-C*_*Lp(a)corM/H*_ Corrected for Lp(a) levels low-density lipoprotein cholesterol as calculated by the Marin/Hopkins formula, dLDL_F-M/H_: LDL-C_F_ - LDL-C_MH_, dLDL_F-Lp(a)corM/H_: LDL-C_F_ - LDL_Lp(a)corM/H_

^*^P < 0.05 for the difference between LDL-C formulas in each quartile

**Fig. 2:**
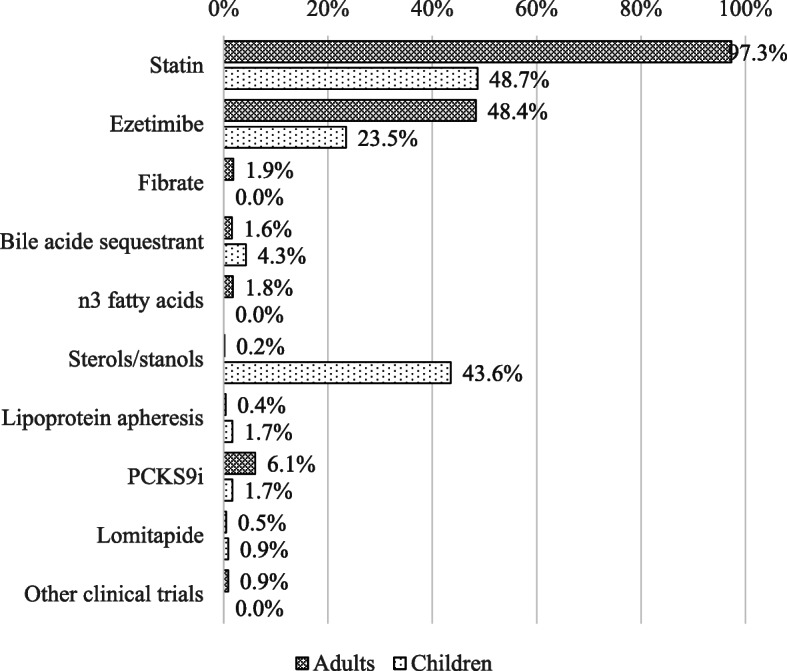
Type of lipid-lowering treatment

Among adult patients, a total of 67.9% (*n* = 966) were on lipid-lowering treatment at the time of the enrollment in HELLAS-FH. Lipid-lowering treatment is presented in Fig. 2 and lipid profile of treated patients in Tables 2 and 4. Mean on-treatment LDL-C_F_ levels [150 ± 71 mg/dL (3.9 ± 1.8 mmol/L)] was similar to the LDL-C_M/H_ levels [151 ± 70 mg/dL (3.9 ± 1.8 mmol/L); *P* = 0.746]. However, mean LDL-C_Lp(a)corM/H_ [121 ± 62 mg/dL (3.1 ± 1.6 mmol/L); *P* < 0.001] was significantly lower compared with LDL-C_F_ levels.

**Table 4:** Lipid profile of adult patients on lipid-lowering treatment

Parameter	Adults			
	Statin ± Ezetimibe (***n*** = 891)		Statin ± Ezetimibe + PCSK9i (***n*** = 59)	
	Pre-treatment	Post-treatment	Pre-treatment	Post-treatment
Total cholesterol, mg/dL (mmol/L)	326 ± 79 (8.4 ± 2.0)	225 ± 63^††^ (5.8 ± 1.6)^††^	392 ± 123 (10.1 ± 3.2)	215 ± 109^††^ (5.6 ± 2.8)
Triglycerides, mg/dL (mmol/L)	128 (94–179) [1.4 (1.4–2.0)]	110 (80–154)^††^ [1.2 (0.9–1.7)]^††^	140 (99–192) [1.6 (1.1–2.2)]	108 (75–153)^††^ [1.2 (0.8–1.7)]^††^
HDL-C, mg/dL (mmol/L)	51 ± 14 (1.3 ± 0.4)	51 ± 15 (1.3 ± 0.4)	51 ± 13 (1.3 ± 0.3)	50 ± 14 (1.3 ± 0.3)
non-HDL-C, mg/dL (mmol/L)	275 ± 79 (7.1 ± 2.0)	174 ± 63^††^ (4.5 ± 1.6)^††^	342 ± 119 (8.8 ± 3.1)	164 ± 106^††^ (4.2 ± 2.7)^††^
LDL-C_F_, mg/dL (mmol/L)	244 ± 68 (6.3 ± 1.8)	149 ± 59^††^ (3.9 ± 1.5)^††^	311 ± 118 (8.0 ± 3.1)	141 ± 103^††^ (3.6 ± 2.7)^††^
LDL-C_M/H_, mg/dL (mmol/L)	244 ± 68 (6.3 ± 1.8)	150 ± 59^††^ (3.9 ± 1.5)^††^	311 ± 118 (8.0 ± 3.1)	141 ± 102^††^ (3.6 ± 2.6)^††^
^a^LDL-C_Lp(a)corM/H_, mg/dL (mmol/L)	243 ± 77 (6.3 ± 2.0)	118 ± 52^†,§,††^ (3.1–1.3)^†,§,††^	-^b^	-^b^
^a^Lp(a), mg/dL (nmol/L)	25 (10–68) [50.7 (18.0–144)]	26 (10–68) [52.9 (18.0–144)]	-^b^	-^b^

Data are presented as mean ± standard deviation or median (interquartile range) for parametric and non-parametric variables, respectively

*HDL-C* High-density lipoprotein cholesterol, *LDL-C*_*F*_ Low-density lipoprotein cholesterol as calculated by the Friedewald formula, *LDL-C*_*M/H*_ Low-density lipoprotein cholesterol as calculated by the Marin/Hopkins formula, *LDL-C*_*Lp(a)corM/H*_ Corrected for Lp(a) levels low-density lipoprotein cholesterol as calculated by the Marin/Hopkins formula, *PCKS9i* Proprotein convertase subtilisin/kexin type 9 inhibitor, Lp(a) was converted using the formula: Lp(a) nmol/L = 2.18 × Lp(a) mg/dL − 3.83

^†^*P* < 0.001 vs LDL-C_F_ and LDL-C_M/H_, ^§^*P* < 0.001 for the comparison of LDL-C change compared with the change of LDL-C_F_ and LDL-C_M/H,_^††^*P* < 0.001 vs pre-treatment

^a^: Data available for 342 patients

^b^: Data for Lp(a) levels in adult patients treated with PCSK9i were available only for 5 patients and thus were not included in the analysis

Pearson’s correlation showed significant correlation between post-treatment LDL-C_F_ and LDL-C_M/H_ (*r* = 0.994, *P* < 0.001, Fig. 1c) as well as between LDL-C_F_ and LDL-C_Lp(a)corM/H_ (*r* = 0.951, *P* < 0.001, Fig. 1d).

In Table 4 we evaluate the effects on lipid profile of statin ± ezetimibe vs statin ± ezetimibe + PCSK9i treatment regimens (Table 4). We found that levels of Lp(a) did not significantly change in the statin ± ezetimibe group. Unfortunately, in the subset of patients on PCSK9i treatment (*n* = 59) we had available Lp(a) data for only 5 patients and therefore no meaningful analysis for LDL-C_Lp(a)corM/H_ could be performed. The differences of LDL-C assessment between the Friedewald and the Martin/Hopkins methods were similar in both the statin ± ezetimibe and the statin ± ezetimibe + PCSK9i group (data not shown).

When we compared treated patients with TG ≥150 mg/dL (≥1.7 mmol/L) (*n* = 264), LDL-C_M/H_ levels tended to be higher compared with the LDL-C_F_ [174 ± 81 mg/dL (4.5 ± 2.1 mmol/L) vs 169 ± 84 mg/dL (4.4 ± 2.2 mmol/L), respectively; *P* = 0.38]. No difference was observed between the LDL-C_M/H_ and LDL-C_F_ concentrations in treated patients with TG < 150 mg/dL (< 1.7 mmol/L) [142 ± 63 mg/dL (3.7 ± 1.6 mmol/L) vs 143 ± 64 mg/dL (3.7 ± 1.7 mmol/L), respectively; *P* = 0.770]. Moreover, in treated patients with LDL-C_F_ levels ≤70 mg/dL (< 1.8 mmol/L) (*n* = 57), LDL-C_M/H_ levels tended to be higher compared with LDL-C_F_ [60 ± 14 mg/dL (1.6 ± 0.4 mmol/L) vs 56 ± 14 mg/dL (1.5 ± 0.4 mmol/L), respectively; *P* = 0.158]. No significant difference was observed between LDL-C_M/H_ and LDL-C_F_ levels in those with LDL-C_F_ levels > 70 mg/dL (> 1.8 mmol/L) (157 ± 68 mg/dL (4.1 ± 1.8 mmol/L) vs 157 ± 69 (4.1 ± 1.8 mmol/L) mg/dL, respectively; *P* = 0.791). When LDL-C_F_ and LDL-C_M/H_ levels were compared in treated patients with both TG ≥150 mg/dL (≥1.7 mmol/L) and LDL-C_F_ ≤ 70 mg/dL (< 1.8 mmol/L), LDL-C_M/H_ levels clearly tended to be higher [70 ± 19 mg/dL (1.8 ± 0.5 mmol/L) vs 51 ± 19 mg/dL (1.3 ± 0.5 mmol/L), respectively; *P* = 0.099] in contrast to treated patients with both TG < 150 mg/dL (< 1.7 mmol/L) and LDL-C_F_ > 70 mg/dL (> 1.8 mmol/L) (149 ± 61 mg/dL (3.9 ± 1.6 mmol/L) vs 150 ± 62 mg/dL (3.9 ± 1.6 mmol/L), respectively; *P* = 0.739). Of note, in treated patients with LDL-C_F_ ≤ 70 mg/dL (≤1.8 mmol/L) (*n* = 57) a total of 14% (*n* = 8) had LDL-C_M/H_ > 70 mg/dL (> 1.8 mmol/L). Similarly, in treated patients with LDL-C_F_ ≤ 100 mg/dL (≤2.6 mmol/L) (*n* = 208), a total of 6.7% (*n* = 14) had LDL-C_M/H_ > 100 mg/dL (> 2.6 mmol/L).

We dichotomized treated patients based on whether they had Lp(a) levels < 50 mg/dL (< 105 nmol/L) or ≥ 50 mg/dL (≥ 105 nmol/L). LDL-C_F_ was similar compared with the LDL-C_Lp(a)corM/H_ [132 ± 59 (3.4 ± 1.5 mmol/L) vs 128 ± 58 (3.3 ± 1.5 mmol/L) mg/dL, respectively; *P* = 0.554] in the Lp(a) < 50 mg/dL (< 105 nmol/L) group, but significantly higher [139 ± 67 mg/dL (3.6 ± 1.7 mmol/L) vs 108 ± 68 (2.8 ± 1.8 mmol/L) mg/dL, respectively; *P* = 0.002] in the Lp(a) ≥50 mg/dL (≥105 nmol/L) group.

**Fig. 3:**
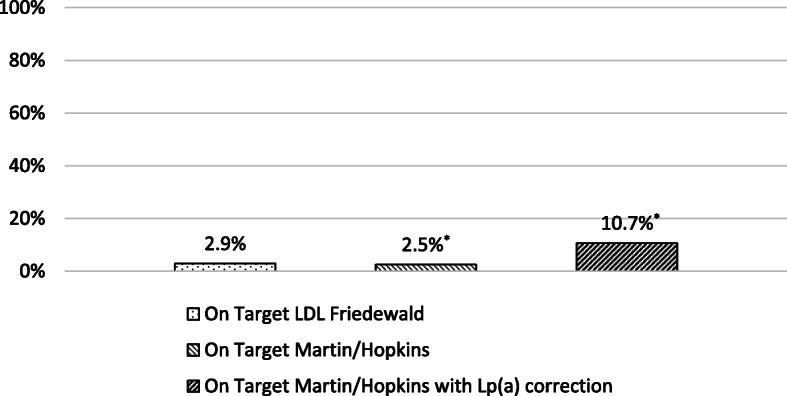
Percentage of low-density lipoprotein cholesterol target achievement in adults. ^*^: *P* < 0.001 vs LDL-C_F_

A total of 2.9% of treated patients reached their LDL-C target [< 70 mg/dL (1.8 mmol/L) or < 55 mg/dL (1.4 mmol/L) on an individual basis] when the Friedewald formula was used (Fig. 3). This percentage was significantly lower (2.5%) with the Martin/Hopkins equation (*P* < 0.001). After correcting the LDL-C_M/H_ calculation for Lp(a) levels the percentage of patients achieving their LDL-C targets was even higher (10.7%; *P* < 0.001 for the comparison with both with Friedewald and Martin/Hopkins formulas).

**Fig. 4:**
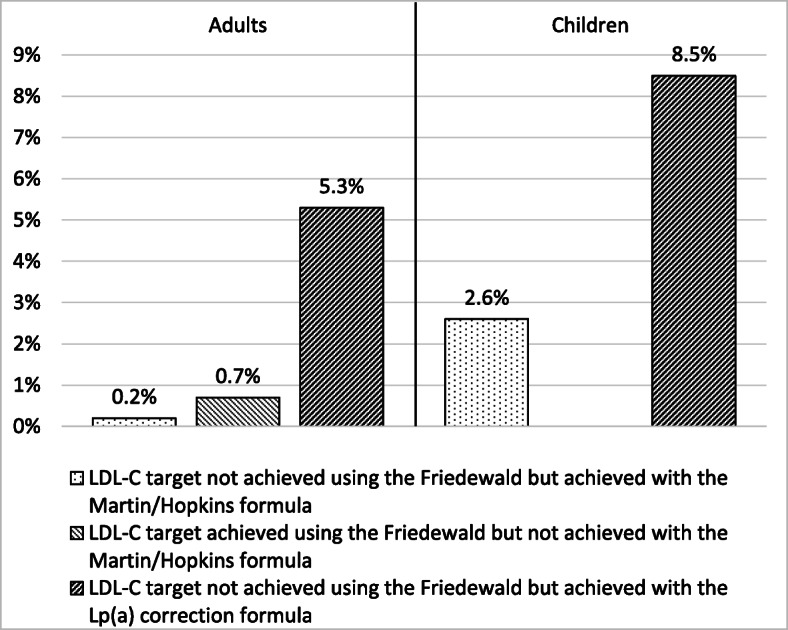
Reclassification of low-density lipoprotein cholesterol (LDL-C) target achievement in familial hypercholesterolemia patients with the Martin/Hopkins and the Martin/Hopkins adjusted for lipoprotein (a) levels formulas for the calculation of LDL-C compared with the Friedewald formula

Among treated patients, a total of 0.2% (*n* = 2) that had been classified as not achieving LDL-C target when Friedewald formula was used, was reclassified as reaching target when Martin/Hopkins equation was applied (Fig. 4). Conversely, a total of 0.7% of patients (*n* = 7) that had been classified as achieving LDL-C target with Friedewald formula was reclassified as not reaching target with the Martin/Hopkins equation (Fig. 4). Moreover, a total of 5.3% (*n* = 19) of treated patients that were not achieving LDL-C target when assessed by the Friedewald formula, was reclassified as reaching target when the Lp(a) correction formula was used (Fig. 4).

### Children subgroup (< 18 years)

A total of 197 children (100 boys) were included in the analysis. Their mean age at the time of enrolment was 11.0 ± 3.5 years (7.3 ± 3.9 years at the time of FH diagnosis). Baseline demographic characteristics and lipid profiles are presented in Tables 1 and 2, respectively.

At diagnosis LDL-C_F_ and LDL-C_M/H_ levels were similar [233 ± 65 mg/dL (6.0 ± 1.7 mmol/L] vs 229 ± 65 mg/dL (5.9 ± 1.7 mmol/L), respectively; *P* = 0.606]. Lp(a) levels were available in 95 children both at diagnosis and on-treatment. At diagnosis LDL-C_Lp(a)corM/H_ levels were numerically lower [LDL-C_Lp(a)corM/H_ 225 ± 70 mg/dL (5.8 ± 1.8 mmol/L)] compared with LDL-C_F_, but this difference did not reach significance (*P* = 0.311). Pearson’s correlation showed significant correlation between LDL-C_F_ and LDL-C_M/H_ (*r* = 0.998, *P* < 0.001, Fig. 1e) as well as between LDL-C_F_ and LDL-C_Lp(a)corM/H_ (*r* = 0.974, *P* < 0.001, Fig. 1f). Moreover, patients were split in quartiles based on baseline LDL-C_F_ and the differences of LDL-C in each quartile were then compared across the 3 methods (Table 3). Median LDL-C_M/H_ and LDL-C_Lp(a)corM/H_ were significantly higher compared with the LDL-C_F_) in all but the first quartiles.

A total of 59.4% children (*n* = 117) were on lipid-lowering therapy at the time of the enrollment. LDL-C_F_ levels [183 ± 97 mg/dL (4.7 ± 2.5 mmol/L)] were similar to LDL-C_M/H_ levels [180 ± 97 mg/dL (4.7 ± 2.5 mmol/L); *P* = 0.820] as well as LDL-C_Lp(a)-corM/H_ levels [174 ± 94 mg/dL (4.5 ± 2.4 mmol/L); *P* = 0.503]. In treated children Pearson’s correlation showed significant correlation between LDL-C_F_ and LDL-C_M/H_ (*r* = 0.998, *P* < 0.001, Fig. 1g) as well as between LDL-C_F_ and LDL-C_Lp(a)corM/H_ (*r* = 0.987, *P* < 0.001, Fig. 1h). Lp(a) levels did not significantly change in the statin ± ezetimibe group (Table 5).

**Table 5:** Lipid profile of children on lipid-lowering treatment

Parameter	Children	
	Statin ± Ezetimibe (***n*** = 57)	
	Pre-treatment	Post-treatment
Total cholesterol, mg/dL (mmol/L)	322 ± 81 (8.3 ± 2.1)	233 ± 133^§^ (6.0 ± 3.4)^§^
Triglycerides, mg/dL (mmol/L)	77 (53–105) [0.9 (0.6–1.2)]	66 (53–89) [0.7 (0.6–1.0)]
HDL-C, mg/dL (mmol/L)	54 ± 14 (1.4 ± 0.4)	52 ± 12 (1.3 ± 0.3)
non-HDL-C, mg/dL (mmol/L)	268 ± 81 (6.9 ± 2.1)	180 ± 136^§^ (4.7 ± 35) ^§^
LDL-C_F_, mg/dL (mmol/L)	251 ± 79 (6.5 ± 2.0)	148 ± 58^§^ (3.8 ± 1.5) ^§^
LDL-C_M/H_, mg/dL (mmol/L)	248 ± 79 (6.4 ± 2.0)	146 ± 57^§^ (3.8 ± 1.5)^§^
^a^LDL-C_Lp(a)corM/H_, mg/dL (mmol/L)	253 ± 88 (6.5 ± 2.3)	125 ± 54^†,§,§§^ (3.2 ± 1.4)^§,§§^
^a^Lp(a), mg/dL (nmol/L)	21 (6–86) [42.0 (9.3–183.7)]	21 (9–61) [42.0 (15.8–129.2)]

Data are presented as mean ± standard deviation or median (interquartile range) for parametric and non-parametric variables, respectively

*HDL-C* High-density lipoprotein cholesterol, *LDL-C*_*F*_ Low-density lipoprotein cholesterol as calculated by the Friedewald formula, *LDL-C*_*M/H*_ Low-density lipoprotein cholesterol as calculated by the Marin/Hopkins formula, *LDL-C*_*Lp(a)corM/H*_ Corrected for Lp(a) levels low-density lipoprotein cholesterol as calculated by the Marin/Hopkins formula, *PCKS9i* Proprotein convertase subtilisin/kexin type 9 inhibitor, Lp(a) was converted using the formula: Lp(a) nmol/L = 2.18 × Lp(a) mg/dL − 3.83

^†^: *P* < 0.001 vs LDL-C_F_, ^§^*P* < 0.001 vs pre-treatment, ^§§^*P* < 0.001 for the comparison of LDL-C change compared with the change of LDL-C_F_ and LDL-C_M/H_

^a^: Data available for 40 patients

**Fig. 5:**
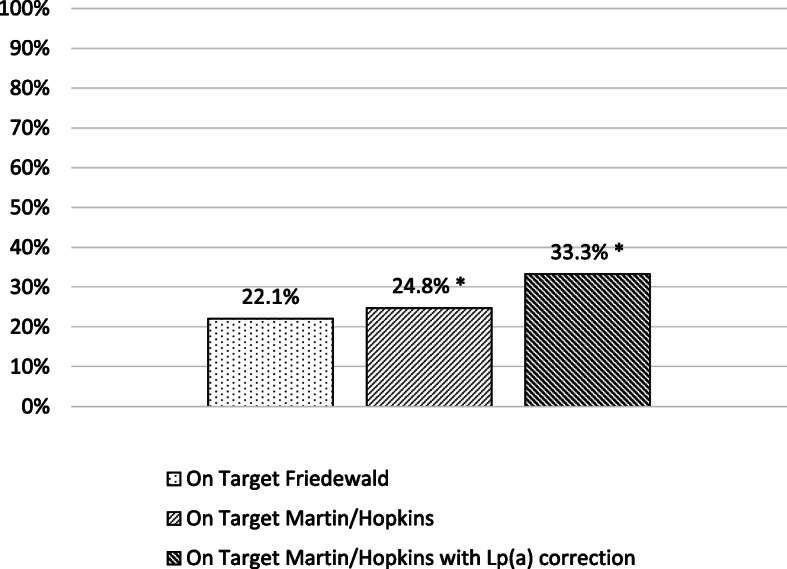
Percentage of low-density lipoprotein cholesterol target achievement in children. ^*^: *P* < 0.001 vs LDL-C_F_

A total of 22.1% of the treated children reached LDL-C target [≤130 mg/dL (3.4 mmol/L)] when LDL-C was calculated by the Friedewald formula (Fig. 5). This percentage was 24.8% when using Martin/Hopkins equation (*P* < 0.001) and 33.3% when correcting LDL-C_M/H_ for Lp(a) (*P* < 0.001 vs Friedewald formula).

Among treated children, a total of 2.6% (*n* = 3) that had been classified as not achieving LDL-C target with Friedewald formula, was reclassified as reaching target with Martin/Hopkins equation. Moreover, a total of 8.5% (*n* = 10) of treated patients that were not achieving LDL-C target when assessed by Friedewald formula were reclassified as reaching target with the Lp(a) correction formula (Fig. 4).

## Discussion

In the present study, LDL-C levels were calculated and compared using the Friedewald and Martin/Hopkins equations as well as after correcting for Lp(a) concentrations in 1620 patients participating in the HELLAS-FH registry. Similar values were observed for LDL-C_F_ and LDL-C_M/H_ at diagnosis and on-treatment in adult patients (Table 2). There was a trend for lower LDL-C_Lp(a)corM/H_ levels at diagnosis in adult patients, whereas on-treatment LDL-C_Lp(a)corM/H_ was significantly lower compared with on-treatment LDL-C_F_. Accordingly, target achievement rate in adults was lower with LDL-C_M/H_ and higher with LDL-C_Lp(a)-corM/H_ as compared with LDL-C_F_. Of note, the present study, to our knowledge, is the first comparing Friedewald formula with Martin/Hopkins and Lp(a)-corrected equations in children. Friedewald and Martin/Hopkins performed similarly both at diagnosis and on treatment. On the other hand, more children achieved LDL-C goal when using corrected for Lp(a) LDL-C_M/H_ compared with the Friedewald formula.

Although the Friedewald formula provides a simple method for calculating LDL-C it comes with the inherent limitation of a fixed value of 5 for the ratio of TG:VLDL-C in all individuals [9]. In this context, the Friedewald formula is not valid for patients with TGs > 400 mg/dL (4.5 mmol/L) and those with dysbetalipoproteinemia [25, 26]. This limitation becomes more pronounced at lower LDL-C levels, which are often seen after the introduction of PCSK9 inhibition [27]. A recent study included 70,209 baseline and on-treatment lipid data from the VOYAGER (an indiVidual patient data meta-analysis Of statin therapY in At risk Groups: Effects of Rosuvastatin, atorvastatin, and simvastatin) trial [28]. Friedewald equation underestimated LDL-C values compared with Martin/Hopkins equation, particularly in low LDL-C levels [28]. This could result in undertreatment of some patients [28]. These findings agree with our results, as in patients with both TGs ≥150 mg/dL (≥ 1.7 mmol/L) and LDL-C ≤ 70 mg/dL (1.8 mmol/L) the LDL-C_M/H_ levels had a clear trend for being higher compared with LDL-C_F_.

A recent analysis from the Further Cardiovascular Outcomes Research with PCSK9 Inhibition in patients with Elevated Risk (FOURIER) trial evaluated the accuracy of Martin/Hopkins and Friedewald equations for estimating LDL-C levels compared with preparative ultracentrifugation (PUC) [29]. A total of 56,624 observations from 12,742 patients had Friedewald, Martin/Hopkins and PUC LDL-C measurements. Overall, the correlation with PUC LDL-C was significantly higher for Martin/Hopkins vs Friedewald equation [*r* = 0.918 (95% CI 0.916–0.919) vs *r* = 0.867 (0.865–0.869), *P* < 0.001] [29]. Underestimation of LDL-C_F_ may compromise optimal patient care due to inappropriate withholding, termination or down titration of hypolipidemic therapy.

In the present study, at diagnosis and on-treatment LDL-C_M/H_ was similar compared with LDL-C_F_. Median TG levels at diagnosis and on treatment were 130 (97–181) mg/dL [1.5 (1.1–2.0 mmol/L)] and 111 (80–157) mg/dL [1.3 (0.9–1.8 mmol/L)], respectively. Also, only a very small percentage of patients achieved target LDL-C_F_. Therefore, it is evident that FH patients are undertreated as is often the case in clinical practice [30, 31]. Moreover, since FH patients should be treated to low LDL-C levels, the Martin/Hopkins formula may be preferred given its superior accuracy in low LDL-C compared with the Friedewald formula. Indeed, the recent consensus-based recommendations from EAS and European Federation of Clinical Chemistry and Laboratory Medicine (EFLM) for quantifying atherogenic lipoproteins state that the Martin-Hopkins modified equation may be preferable for calculation of LDL-C [32].

Elevated Lp(a) concentration is a strong risk factor for CVD independent of LDL-C [33]. None of the methods used for LDL-C assessment can separate LDL-C from Lp(a)-C. Calculated LDL-C was shown to be overestimated in patients with high Lp(a) levels, particularly in those with extreme Lp(a) concentrations [34]. Using the Dahlen formula, there is a strong positive correlation between Lp(a) levels and the percentage of LDL-C levels overestimation [34, 35]. Since Lp(a) concentrations are frequently high in patients with FH [36], LDL-C levels are probably overestimated in a considerable number of FH patients. Indeed, in our study a total of 5.3 and 8.5% of adults and children, respectively, that were not achieving their LDL-C_F_ target were reclassified as reaching target after Lp(a) correction. Overall, more patients attained their LDL-C target with Lp(a) correction formula (10.7% in adults and 33.3% in children) compared with Friedewald (2.9% in adults and 22.1% in children) or Martin/Hopkins (2.5% in adults and 24.8% in children) eqs. FH patients with increased Lp(a) levels may require even more aggressive LDL-C reduction [33, 37–40]. Indeed, in the Copenhagen General Population study with 46,200 individuals, the risk of myocardial infarction was the highest in FH patients with Lp(a) > 50 mg/dL (105 nmol/L) [hazard ratio (HR) = 5.3, 95% CI 3.6–7.6], followed by FH and Lp(a) values ≤50 mg/dL (≤105 nmol/L) (HR = 3.2, 95% CI 2.5–4.1) compared with the reference group of subjects without FH and Lp(a) values ≤50 mg/dL (≤105 nmol/L) [41].

Concerns have been raised regarding the possible deleterious effects of statin treatment on Lp(a) levels [42] although no clear consensus on the effects of statins on Lp(a) has been reached as conflicting effects have been observed [43–45]. In our study we observed no increase in Lp(a) levels associated with statin use. Proprotein convertase subtilisin/kexin type 9 (PCSK9) inhibitors and other novel therapies that reduce Lp(a) may contribute to reduction in CVD [46–48]. Indeed, a recent pre-specified analysis of the placebo-controlled ODYSSEY Outcomes (Evaluation of Cardiovascular Outcomes After an Acute Coronary Syndrome During Treatment With Alirocumab) trial evaluated the effects of alirocumab on Lp(a) and Lp(a) corrected LDL-C on CVD risk [49]. In patients with recent acute coronary syndrome the reductions of Lp(a) and Lp(a) corrected LDL-C by alirocumab were independently associated with the absolute reduction in risk of major adverse cardiovascular events. This effect was more pronounced in patients at the highest Lp(a) quartile. This study also highlights the role of Lp(a)-corrected LDL-C as a predictor of recurrent events in the population of ODYSSEY OUTCOMES.

The recent consensus-based recommendations from EAS/EFLM state that Lp(a)-C correction of measured or calculated LDL-C should be applied in patients with known or suspected high Lp(a) concentration, or if the patient shows a poor response to LDL-lowering therapy [32]. Similarly, the latest ESC/EAS guidelines suggest that Lp(a) should be measured at least once in a person’s lifetime [19].

### Study limitations

The main limitation of this study is the lack of direct LDL-C measurement by ultracentrifugation. This was not feasible because data were retrieved from patient files as is the case with most Registries. However, even direct LDL-C may not differentiate ‘true’ LDL-C from Lp(a)-C, as discussed above. The measurement of biochemical parameters was performed at local laboratories instead of a central laboratory. It should be noted, however, that all Registry sites are based in major hospitals, in which laboratories are vigorously calibrated and monitored and Lp(a) was measured by the same method. Unfortunately, no direct comparison of different laboratories was performed. Also, Lp(a) levels were available in a limited number of patients (*n* = 355 for adults and *n* = 95 for children). non-HDL-C can provide a measure of cholesterol content of a wider range of atherogenic lipoproteins than LDL-C. Therefore, the clinical impact of adjustments of calculated LDL-C is questionable.

## Conclusions

Friedewald and Martin/Hopkins equations for LDL-C assessment appear to perform similarly in adult FH patients, except in the case of LDL-C < 70 mg/dL (1.8 mmol/L) and TGs > 150 mg/dL (1.7 mmol/L) where LDL-C_M/H_ is higher compared with LDL-C_F_. On the other hand, the correction of LDL-C for Lp(a)-C levels may result in significantly lower calculated LDL-C concentrations and thus greater LDL-C target achievement rates. In FH children, all 3 LDL-C calculating formulas resulted in similar LDL-C concentrations both at diagnosis and on-treatment, whereas target achievement was higher both with LDL-C_M/H_ and LDL-C_Lp(a)-corM/H_ methods compared with LDL-C_F_. Studies specifically designed to assess the prognostic value of LDL-C as calculated by the Friedewald, Martin/Hopkins or the Lp(a) corrected LDL-C with regard of hard clinical endpoints are required. These studies should research whether the differences among the 3 methods of LDL-C estimation translate in different CVD stratification in both the general population and FH patients. Mendelian randomization studies could provide further insight regarding the performance of the 3 formulas. An important initial step would be to retrospectively re-analyze data from major clinical trial to assess whether LDL-C_M/H_ and/or LDL-C_Lp(a)-corM/H_ perform better in predicting CVD events compared with LDL-C_F_. Moreover, as the HELLAS-FH registry is running, we plan an update when enough prospective data have been gathered in order to validate the 3 different LDL-C estimation methods in the context of CVD events and mortality prognosis.

Our clinical recommendation is that in FH patients both calculation of LDL-C_M/H_ (especially in the case of high TGs and/or very low LDL-C_F_) and Lp(a) measurement should be performed. The LDL-C_M/H_ should then be corrected for Lp(a) levels so as to better estimate ‘true’ LDL-C levels. All in all, the Lp(a)-corrected LDL-C_M/H_, if validated in clinical trials, may prove to be the most meaningful estimation, especially in adults and children with high Lp(a) levels such as those with FH.

## Data Availability

The datasets generated and/or analysed during the current study are not publicly available due to restrictions associated with anonymity of participants but are available from the corresponding author on reasonable request.

## Abbreviations

DLCN: Dutch Lipid Clinic Network
EAS: European Atherosclerosis Society
EFLM: European Federation of Clinical Chemistry and Laboratory Medicine
ESC: European Society of Cardiology
FH: Familial hypercholesterolemia
FOURIER: Further cardiovascular outcomes research with PCSK9 inhibition in patients with elevated risk
HELLAS-FH: Hellenic familial hypercholesterolemia registry
HR: Hazard ratio
IQR: Interquartile range
LDL-C: Low-density lipoprotein cholesterol
LDL-C_F_: Low-density lipoprotein cholesterol calculated using the Friedewald formula
LDL-C_Lp(a)corM/H_: Low-density lipoprotein cholesterol calculated using the Martin/Hopkins formula after correcting for lipoprotein (a)levels
LDL-C_M/H_: Low-density lipoprotein cholesterol calculated using the Martin/Hopkins formula
Lp(a): Lipoprotein(a)
Lp(a)-C: Cholesterol of lipoprotein(a)
non-HDL-C: Non-high density lipoprotein cholesterol
PCSK9: Proprotein convertase subtilisin/kexin type 9
PUC: Preparative ultracentrifugation
SPSS: Statistical Package for the Social Sciences
TCHOL: Total cholesterol
TG: Triglycerides
VLDL-C: Very low-density lipoprotein cholesterol
VOYAGER: An individual patient data meta-analysis of statin therapy in at risk groups: effects of rosuvastatin, atorvastatin, and simvastatin

## References

[1] Nordestgaard BG, Chapman MJ, Humphries SE, Ginsberg HN, Masana L, Descamps OS, Wiklund O, Hegele RA, Raal FJ, Defesche JC (2013). Familial hypercholesterolaemia is underdiagnosed and undertreated in the general population: guidance for clinicians to prevent coronary heart disease: consensus statement of the European Atherosclerosis Society. Eur Heart J.

[2] Reiner Z, Simental-Mendia LE, Ruscica M, Katsiki N, Banach M, Al Rasadi K, Jamialahmadi T, Sahebkar A (2019). Pulse wave velocity as a measure of arterial stiffness in patients with familial hypercholesterolemia: a systematic review and meta-analysis. Arch Med Sci.

[3] Miname MH, Santos RD (2019). Reducing cardiovascular risk in patients with familial hypercholesterolemia: risk prediction and lipid management. Prog Cardiovasc Dis.

[4] Dyrbus K, Gasior M, Desperak P, Osadnik T, Nowak J, Banach M (2019). The prevalence and management of familial hypercholesterolemia in patients with acute coronary syndrome in the Polish tertiary centre: results from the TERCET registry with 19,781 individuals. Atherosclerosis.

[5] Bhagat U, Das UN (2015). Potential role of dietary lipids in the prophylaxis of some clinical conditions. Arch Med Sci.

[6] Das UN (2008). Essential fatty acids and their metabolites could function as endogenous HMG-CoA reductase and ACE enzyme inhibitors, anti-arrhythmic, anti-hypertensive, anti-atherosclerotic, anti-inflammatory, cytoprotective, and cardioprotective molecules. Lipids Health Dis.

[7] Raal FJ, Hovingh GK, Catapano AL (2018). Familial hypercholesterolemia treatments: guidelines and new therapies. Atherosclerosis.

[8] Ito MK, Watts GF (2015). Challenges in the diagnosis and treatment of homozygous familial hypercholesterolemia. Drugs.

[9] Friedewald WT, Levy RI, Fredrickson DS (1972). Estimation of the concentration of low-density lipoprotein cholesterol in plasma, without use of the preparative ultracentrifuge. Clin Chem.

[10] DeLong DM, DeLong ER, Wood PD, Lippel K, Rifkind BM (1986). A comparison of methods for the estimation of plasma low- and very low-density lipoprotein cholesterol. The lipid research clinics prevalence study. JAMA.

[11] Martin SS, Blaha MJ, Elshazly MB, Toth PP, Kwiterovich PO, Blumenthal RS, Jones SR (2013). Comparison of a novel method vs the Friedewald equation for estimating low-density lipoprotein cholesterol levels from the standard lipid profile. JAMA.

[12] Yeang C, Witztum JL, Tsimikas S (2015). ‘LDL-C’ = LDL-C + Lp(a)-C: implications of achieved ultra-low LDL-C levels in the proprotein convertase subtilisin/kexin type 9 era of potent LDL-C lowering. Curr Opin Lipidol.

[13] Kinpara K, Okada H, Yoneyama A, Okubo M, Murase T (2011). Lipoprotein(a)-cholesterol: a significant component of serum cholesterol. Clin Chim Acta.

[14] Marcovina SM, Albers JJ, Scanu AM, Kennedy H, Giaculli F, Berg K, Couderc R, Dati F, Rifai N, Sakurabayashi I (2000). Use of a reference material proposed by the International Federation of Clinical Chemistry and Laboratory Medicine to evaluate analytical methods for the determination of plasma lipoprotein(a). Clin Chem.

[15] Viney NJ, Yeang C, Yang X, Xia S, Witztum JL, Tsimikas S (2018). Relationship between “LDL-C”, estimated true LDL-C, apolipoprotein B-100, and PCSK9 levels following lipoprotein(a) lowering with an antisense oligonucleotide. J Clin Lipidol.

[16] Gaubatz JW, Heideman C, Gotto AM, Morrisett JD, Dahlen GH (1983). Human plasma lipoprotein [a]. Structural properties. J Biol Chem.

[17] Faghihnia N, Tsimikas S, Miller ER, Witztum JL, Krauss RM (2010). Changes in lipoprotein(a), oxidized phospholipids, and LDL subclasses with a low-fat high-carbohydrate diet. J Lipid Res.

[18] Dahlen GH, Scanu AM (1990). Incidence of Lp(a) among populations. Lipoprotein(a).

[19] Mach F, Baigent C, Catapano AL, Koskinas KC, Casula M, Badimon L, Chapman MJ, De Backer GG, Delgado V, Ference BA (2020). 2019 ESC/EAS guidelines for the management of dyslipidaemias: lipid modification to reduce cardiovascular risk. Eur Heart J.

[20] Rizos CV, Athyros V, Bilianou E, Chrousos G, Garoufi A, Kolovou G, Kotsis V, Rallidis L, Skalidis E, Skoumas I (2017). An insight into familial hypercholesterolemia in Greece: rationale and design of the Hellenic Familial Hypercholesterolemia Registry (HELLAS-FH). Hormones (Athens).

[21] Rizos CV, Elisaf MS, Skoumas I, Tziomalos K, Kotsis V, Rallidis L, Garoufi A, Athyros VG, Skalidis E, Kolovou G (2018). Characteristics and management of 1093 patients with clinical diagnosis of familial hypercholesterolemia in Greece: data from the Hellenic Familial Hypercholesterolemia Registry (HELLAS-FH). Atherosclerosis.

[22] Civeira F, Ros E, Jarauta E, Plana N, Zambon D, Puzo J, Martinez de Esteban JP, Ferrando J, Zabala S, Almagro F (2008). Comparison of genetic versus clinical diagnosis in familial hypercholesterolemia. Am J Cardiol.

[23] Goldberg AC, Hopkins PN, Toth PP, Ballantyne CM, Rader DJ, Robinson JG, Daniels SR, Gidding SS, de Ferranti SD, Ito MK (2011). Familial hypercholesterolemia: screening, diagnosis and management of pediatric and adult patients: clinical guidance from the National Lipid Association Expert Panel on Familial Hypercholesterolemia. J Clin Lipidol.

[24] Wiegman A, Gidding SS, Watts GF, Chapman MJ, Ginsberg HN, Cuchel M, Ose L, Averna M, Boileau C, Borén J (2015). Familial hypercholesterolaemia in children and adolescents: gaining decades of life by optimizing detection and treatment. Eur Heart J.

[25] Cordova CM, Schneider CR, Juttel ID, Cordova MM (2004). Comparison of LDL-cholesterol direct measurement with the estimate using the Friedewald formula in a sample of 10,664 patients. Arq Bras Cardiol.

[26] McNamara JR, Cohn JS, Wilson PW, Schaefer EJ (1990). Calculated values for low-density lipoprotein cholesterol in the assessment of lipid abnormalities and coronary disease risk. Clin Chem.

[27] Quispe R, Hendrani A, Elshazly MB, Michos ED, McEvoy JW, Blaha MJ, Banach M, Kulkarni KR, Toth PP, Coresh J (2017). Accuracy of low-density lipoprotein cholesterol estimation at very low levels. BMC Med.

[28] Palmer MK, Barter PJ, Lundman P, Nicholls SJ, Toth PP, Karlson BW (2019). Comparing a novel equation for calculating low-density lipoprotein cholesterol with the Friedewald equation: a VOYAGER analysis. Clin Biochem.

[29] Martin SS, Giugliano RP, Murphy SA, Wasserman SM, Stein EA, Ceska R, Lopez-Miranda J, Georgiev B, Lorenzatti AJ, Tikkanen MJ (2018). Comparison of low-density lipoprotein cholesterol assessment by Martin/Hopkins estimation, Friedewald estimation, and preparative ultracentrifugation: insights from the FOURIER trial. JAMA Cardiol.

[30] Toth PP, Granowitz C, Hull M, Anderson A, Philip S (2019). Long-term statin persistence is poor among high-risk patients with dyslipidemia: a real-world administrative claims analysis. Lipids Health Dis.

[31] Rizos CV, Barkas F, Elisaf MS (2014). Reaching low density lipoprotein cholesterol targets. Curr Med Res Opin.

[32] Langlois MR, Nordestgaard BG, Langsted A, Chapman MJ, Aakre KM, Baum H, Boren J, Bruckert E, Catapano A, Cobbaert C (2020). Quantifying atherogenic lipoproteins for lipid-lowering strategies: consensus-based recommendations from EAS and EFLM. Clin Chem Lab Med.

[33] Nordestgaard BG, Langsted A (2016). Lipoprotein (a) as a cause of cardiovascular disease: insights from epidemiology, genetics, and biology. J Lipid Res.

[34] Saeedi R, Li M, Allard M, Frohlich J (2014). Marked effects of extreme levels of lipoprotein(a) on estimation of low-density lipoprotein cholesterol. Clin Biochem.

[35] Li KM, Wilcken DE, Dudman NP (1994). Effect of serum lipoprotein(a) on estimation of low-density lipoprotein cholesterol by the Friedewald formula. Clin Chem.

[36] Elisaf M, Bairaktari H, Siamopoulos KC (1996). Lp(a) levels in Greek patients with heterozygous familial hypercholesterolemia. Int J Cardiol.

[37] Nordestgaard BG, Chapman MJ, Ray K, Boren J, Andreotti F, Watts GF, Ginsberg H, Amarenco P, Catapano A, Descamps OS (2010). Lipoprotein(a) as a cardiovascular risk factor: current status. Eur Heart J.

[38] Saeedi R, Frohlich J (2016). Lipoprotein (a), an independent cardiovascular risk marker. Clin Diabetes Endocrinol.

[39] Cai A, Li L, Zhang Y, Mo Y, Mai W, Zhou Y (2013). Lipoprotein(a): a promising marker for residual cardiovascular risk assessment. Dis Markers.

[40] Kouvari M, Panagiotakos DB (2019). The role of lipoprotein (a) in primary and secondary cardiovascular disease prevention: a systematic review of epidemiological studies. Curr Opin Cardiol.

[41] Langsted A, Kamstrup PR, Benn M, Tybjaerg-Hansen A, Nordestgaard BG (2016). High lipoprotein(a) as a possible cause of clinical familial hypercholesterolaemia: a prospective cohort study. Lancet Diabetes Endocrinol.

[42] Tsimikas S, Gordts P, Nora C, Yeang C, Witztum JL. Statin therapy increases lipoprotein(a) levels. Eur Heart J. 2019; [Epub ahead of print]. 10.1093/eurheartj/ehz310 31111151

[43] van Wissen S, Smilde TJ, Trip MD, de Boo T, Kastelein JJ, Stalenhoef AF (2003). Long term statin treatment reduces lipoprotein(a) concentrations in heterozygous familial hypercholesterolaemia. Heart.

[44] Willeit P, Ridker PM, Nestel PJ, Simes J, Tonkin AM, Pedersen TR, Schwartz GG, Olsson AG, Colhoun HM, Kronenberg F (2018). Baseline and on-statin treatment lipoprotein(a) levels for prediction of cardiovascular events: individual patient-data meta-analysis of statin outcome trials. Lancet.

[45] Banach M, Penson PE (2020). Statins and Lp(a): do not make perfect the enemy of excellent. Eur Heart J.

[46] O'Donoghue ML, Fazio S, Giugliano RP, Stroes ESG, Kanevsky E, Gouni-Berthold I, Im K, Lira Pineda A, Wasserman SM, Ceska R (2019). Lipoprotein(a), PCSK9 inhibition, and cardiovascular risk. Circulation.

[47] Liberopoulos E. Lipoprotein(a) reduction with PCSK9 inhibitors: an unsolved mystery. Eur J Prev Cardiol. 2020; [Epub ahead of print]. 10.1177/2047487320926777 33611488

[48] Ray KK, Vallejo-Vaz AJ, Ginsberg HN, Davidson MH, Louie MJ, Bujas-Bobanovic M, Minini P, Eckel RH, Cannon CP (2019). Lipoprotein(a) reductions from PCSK9 inhibition and major adverse cardiovascular events: pooled analysis of alirocumab phase 3 trials. Atherosclerosis.

[49] Bittner VA, Szarek M, Aylward PE, Bhatt DL, Diaz R, Edelberg JM, Fras Z, Goodman SG, Halvorsen S, Hanotin C (2020). Effect of Alirocumab on lipoprotein(a) and cardiovascular risk after acute coronary syndrome. J Am Coll Cardiol.

